# Nutrition, supplementation and weight reduction in combat sports: a review

**DOI:** 10.3934/publichealth.2021038

**Published:** 2021-07-06

**Authors:** Paulina Januszko, Ewa Lange

**Affiliations:** Department of Dietetics, Institute of Human Nutrition Sciences, Warsaw University of Life Sciences, SGGW, Warsaw, Poland

**Keywords:** combat sports, nutrition, supplementation, weight reduction

## Abstract

Nutrition is the aspect closely connected to physical activity and may affect body composition, sports performance and post-workout regeneration. Using an appropriate diet plan is a proven method to optimize performance improvements in combat sports. In the majority of combat sports athletes are classified according to their body mass in order to minimize differences between competitors. Many athletes induce weight loss in order to gain an advantage over their opponents. The review was undertaken to provide safe, evidence-based protocols helping athletes in weight reduction without negative effects on sports performance. The nutritional requirements for combat sports athletes, sports supplements, gradual and rapid weight reduction strategies are discussed in this review.

## 1. Introduction

Combat sport is a term used to describe a competitive contact sport where two combatants fight against each other using a particular contest's rules [1]. The most popular combat sports include wrestling, judo, kickboxing, mixed martial arts, boxing, karate and taekwondo. In the majority of combat sports, athletes are classified according to their body mass in order to minimize differences between competitors including body size, strength and agility [2]. Most combat sports may be classified as intermittent, high-intensity sports [3].

There are many methods that have proved to optimize performance improvements including an appropriate diet plan or using sports supplements. Nutrition as the aspect closely connected to physical activity may affect body composition, sports performance and post-workout regeneration. The nutritional requirements for athletes are higher than for the average population for many nutrients. In combat sports the recommended nutrition and energy intake may further exceed levels for non-training population [4].

## 2. Energy and carbohydrates

The primary component to optimize sports performance in combat sports is ensuring an adequate energy intake. The caloric needs for athletes training with moderate or high intensity may approach 40–70 kcals/kg/day depending on the intensity and frequency of trainings. For elite athletes the recommended energy intake may further exceed these levels [5]. It has to be mentioned that the fundamental factor of sports nutrition is nutrient timing and it refers to the time when the body is able to use macronutrients most effectively [6]. The timing of energy intake may enhance muscle protein synthesis, recovery and tissue repair [5].

The primary source of energy for athletes are carbohydrates. Carbohydrates, as energy substrates, can be oxidized via aerobic metabolism (for instance by glycolytic pathway coupled with Krebs cycle and electron transport chain) or converted into lactate via anaerobic metabolism such as anaerobic glycolysis. As a result, adenosine triphosphate is synthesized and the energy is transferred. The ability to maintain exercise is connected with muscle glycogen content [3]. It has been proved that the proper level of carbohydrates in a diet is connected with a higher strength and muscle glycogen storage [7]. Glycogen is a fuel source of glucose, stored in the cytosol of cells. Skeletal muscle cells involve 1–2% of glycogen and liver cells include 5–6% of glycogen respectively. Glycogen stores in muscles and the liver decrease during physical activity. The prolongation and intensification of the activity leads to an overall reduction of glycogen stores [8]. The recommended intake of carbohydrates in an athletes' diet is related to the type and intensity of trainings and should vary between 4–5 g/kg to 8–10 g/kg of body mass. High intensity, endurance sports are related to greater carbohydrate intake [9],[5]. Combat sports include aerobic and anaerobic metabolism during effort and can deplete glycogen storage during training [10]. In high intensity sports, such as combat sports, the intake of carbohydrates may gain 10–12 g/kg of body mass daily [3]. Carbohydrate is the most important macronutrient in pre-exercise meal. It has been proved that ingesting carbohydrates before training improves endurance and increases muscle and liver glycogen [11],[12]. One of the studies has shown that athletes consuming meals 3 hours before training had higher endurance than people training in the fasted state [13]. In another study, the authors investigated that consumption of a high-carbohydrate meal 4 hours before cycling exercise at 70% maximum oxygen intake (VO2 max) significantly increased muscle and liver glycogen. The exercise was taken after an overnight fast and lasted 105 minutes. The result of the meal consumed before exercise was an increased rate of carbohydrate oxidation and utilization of muscle glycogen [14]. It has to be pointed out that there are several types of carbohydrates which are metabolized differently by the body. Differences between simple and complex carbohydrates are related to their ability to be absorbed and used as a source of quick or more gradual energy [4]. It has been proved that the intake of low glycemic index of carbohydrates can increase the durability of training in comparison to high glycemic index products [15]. However, the type, intensity and the length of time between trainings sessions seems to play a critical role. The athletes who train more than once a day with high intensity should choose carbohydrates with high glycemic index in order to accelerate muscle glycogen synthesis before the second training session [9]. The consumption of carbohydrate is crucial after exercise because it enhances the recovery process and restoration of the glycogen depleted during training [3]. High-glycemic products may restore muscle and liver glycogen stores more quickly in comparision to complex carbohydrates [8]. One type of substances used to improve performance during training is maltodextrins. Maltodextrins are saccharide polymers consisting of D-glucose [16]. They are hydrolyzed to free glucose molecules in the the small intestine and due to the low osmolality, they are commonly used as an ingredient of sports drinks [17]. Consuming 30–60 g of high-glycemic index carbohydrates during an exercise lasting more than one hour maintains high rates of carbohydrates, prevents hypoglycemia and can result in performance benefit [3],[18],[19]. It has been scientifically proven that beverages containing more than one source of transportable carbohydrates (for example maltodextrins and fructose) stimulate greater carbohydrates absorption due to additional transport mechanisms. The study by Wallis et al. [16] has shown that in ingestion maltodextrin and fructose during cycling exercise is related with higher oxidation rates than from ingesting maltodextrin alone.

## 3. Protein

The important macronutrient in combat sports is protein. The recommended intake in an athlete's diet is higher in comparison to non-training people in order to ensure regular muscle protein synthesis. Muscle mass loss can have a negative effect on strength, power and performance [20]. The current protein recommendations for athletes is to gain 1.2–2 g/kg of body mass [21]. It has been proved that the requirements for athletes who are reducing weight can be higher and the recommended level of protein intake can gain 1.8–2.7 g/kg of body mass in order to prevent muscle mass loss during energy deficiency. Consumption of an appropriate amount of essential amino acids, particularly leucine, leads to muscle mass synthesis [22]. Essential amino acids cannot be synthetized de novo by the organism and must be supplied by the diet. Ingestion of essential amino acids in free form or as part of a protein bolus of 20–40 g after the exercise is known to stimulate muscle protein synthesis and could increase strength and improve body composition by increasing lean body mass [23]. A proven effect on stimulating muscle protein synthesis consumed after an exercise session refers to, for instance, milk: whey, casein, and soy protein [20],[24]. Each high-quality protein elicits different digestibility, physiological responses, and different muscle retention [24]. Whey and soy protein have rapid digestion which leads to a large increase in the amount of amino acids in blood. On the opposite side, casein precipitates and coagulates in stomach acid and the absorption and digestion is lower [3]. Furthermore, whey and milk protein (containing both whey and casein) are able to stimulate superior muscle protein synthesis than soy or casein [25].

## 4. Fat

The recommended level of fat in athletes' diet is 25–30% of energy intake and the most preferable sources of fat are unsaturated fatty acids, particularly omega-3 polyunsaturated fatty acids [9],[26]. Omega-3 PUFAS are known to reduce oxidative stress and inflammation, lower the risk of cardiovascular disease, reduce muscle soreness and increase muscle protein synthesis [27]. The study by Smith et al. [20] has shown that 8-weeks omega-3 supplementation including 1.86 g EPA and 1.5 g DHA/day increased muscle protein anabolic response to hyperinsulinemia–hyperaminoacidaemia in healthy young adults. Excessive consumption of trans and saturated fatty acids is connected with health implications and should be reduced in dietary intake. The nutritional requirements for fat should be mostly covered by vegetable oils, sea fish, nuts and seeds [9],[26]. In sports, the energy for cellular functions is derived from metabolism glycogen or triglycerides. By the oxidative energy system, the oxygen is utilized in mitochondria of cells resulting in production of ATP [29]. During light and moderate exercises, energy needs of the muscle are met by carbohydrates and free fatty acids [26]. By the lipolysis, triglycerides stored in muscles and adipose sites are metabolized into glycerol and free fatty acids. The maximal fat oxidation can be reached at approximately 47–52% of VO2 max in untrained individuals and 59–64% of VO2 max in endurance-trained athletes [29]. In contrast to carbohydrates, fats are known to have unlimited availability. While increasing the duration of training, the carbohydrates stores are decreasing until they are finally depleted [4].

## 5. Water

Water is a crucial ergogenic aid for athletes and an adequate level of hydration is necessary to maintain exercise capacity [30]. It has been proved that 2% of dehydration can impair sports performance. Dehydration of 4% may lead to serious health outcomes such as heat exhaustion, heat stroke or heat illness [5]. It has been proposed that dehydration used for weight loss increases the risk of acute cardiovascular problems including ischaemic heart disease and stroke. Furthermore, significant levels of dehydration may alter the brain morphology and increase the risk of brain injury related to head trauma induced by strikes [31]. The physical activity induces sweating which is with mineral loss, particularly sodium, potassium, calcium and magnesium [32]. The duration of training, heat acclimatization, weather conditions or genetic predisposition influence the sweating level and determine fluid needs [33]. Recent studies have shown that the type of the training plays an important role in fluid loss. In the study by Rivera-Brown et al. [34] authors indicated that sweat loss and the level of dehydration during training in a tropical climate was lower in intermittent type of sport in comparision to continous training. The appropriate ratio of sodium and potassium is significant for maintaining adequate fluid balance or ensuring proper cardiovascular and nervous system function [32]. The most important role of magnesium and calcium in the context of sports is regulating muscle function, stabilizing enzymic reactions, regulating energy production and facilitating transport of other nutrients [35]. In order to compensate water loss induced by training, athletes should consume approximately 0.5–2 L/hour of water or glucose-electrolyte solutions during exercise. A good strategy to monitor changes in fluid balance is weighing athletes before and after a training session in order to provide the proper level of hydration and increase the sports performance [5]. In the study by Stefanovsky et al. [36] authors have shown that the hydration status in youth judo athletes during an off-season was not optimal. The similar results have been indicated in the research by Petterson and Berg [37] where 89% of combat sports athletes participating in the study were hypohydrated in the morning of the competition day. An effective method for improving short duration performance following acute hypohydration may be passive heat- acclimation protocols [31].

## 6. Supplements

The influence on strength, endurance and body composition may also have sports supplements. There are many substances which have a proven impact on the effectiveness of training [38],[39]. Using supplements may delay the onset of fatigue and allow an athlete to train in higher intensity [3]. According to the Australian Institute of Sports, supplement ingredients are ranked into 4 groups in order to determine if the product is safe and effective at improving sports performance. Substances classificated in group A are scientifically proven to support, and enhance sports performance and prevent or treat clinical issues, for instance sports food, vitamin D, calcium, iron, caffeine or creatine. Substances ranked in group B are based on emerging scientific support and deserving of further research. The substances that have been classificated in group B are for example food polyphenols, fish oils, carnitine, vitamin C and E or Branched-Chain Amino Acids. In group C the supplements are not supportive of benefit amongst athletes and substances classified in the group D are banned to be used by athletes [40]. Substances such as stimulants, prohormones and hormone boosters, GH releasers and peptides are on the World Anti-Doping Agency (WADA) list and shouldn't be injected by athletes [41]. In combat sports, the benefits of using supplements may be provided below two strategies- an acute supplementation and chronic supplementation strategy. An acute supplementation strategy includes the consumption of supplements which have the ability to optimize performance improvements when used in the minutes to hours before training or competition. A chronic supplementation strategy refers to the consumption of sports supplements that need to be used over a period including several days or weeks in order to enhance sports performance [42].

One of the substances which have a proven ergogenic effect in combat sports is caffeine [42]. Caffeine has been ranked in list A of the Australian Institute of Sports [40] and can be found in supplements or food products such as coffee, tea, chocolate or energy drinks [30]. Supplementation with 3–6 mg/kg of caffeine could increase the glycolytic contribution to energy metabolism. The ingestion of caffeine may also improve levels of power, strength and upper arm muscular endurance [43]. The studies have shown that 6 mg/kg of caffeine may decrease extracellular potassium concentration [44]. Ingesting caffeine 60 minutes before training or competition may be adequate due to the plasma levels peak an hour after consuming caffeine [42]. However, it must be noticed that there are some studies showing no ergogenic effect of caffeine supplementation in combat sports [45],[46].

Another ergogenic supplement with evidence-based influence on performance that may be used in combat sports is sodium bicarbonate. Sodium bicarbonate is an alkalizing agent which is able to augment extracellular buffering capacity [42]. During high-intensity exercises, carbon dioxide (CO_2_) and hydrogen (H^+^) cumulate in the blood and muscles. Through the bicarbonate system the body rids itself of the hydrogen and CO_2_ via their conversion to bicarbonate throughout the renal and respiratory system [5]. Sodium bicarbonate has been classified in group A in the list of the Australian Institute of Sports [40]. In the study by Artioli et al. [47] authors have demonstrated that the ingestion 0.3 g/kg of sodium bicarbonate 2 hours before exercise improves performance in specialized judo-tests. Similar effects were shown in the study by Lopes-Silva et al. [48]. Supplementation 300 mg/kg body mass of NaHCO 90 minutes before the combat simulation induced increased lactate concentration and total time attack in taekwondo athletes. Furthermore, greater estimated glycolytic energy contribution in the first round was noticed when compared with placebo. Similar effects may be achieved with a chronic supplementation strategy. One of the studies has shown that ingestion 500 mg of sodium bicarbonate/kg of body mass split in 5 or 4 smaller doses improves work output for two days after cessation of ingestion [49]. Nevertheless, it has to be noticed that supplementing sodium bicarbonate can cause gastrointestinal distress including nausea, diarrhoea, vomiting and stomach pain [46]. The method which is used to minimize the risk of gastrointestinal symptoms is by progressively increasing the doses of sodium bicarbonate. In a study by Durkalec-Michalski, the researchers used a progressive protocol lasting 10 days starting with 0.025 g/kg of body mass on two days and increasing the dose until the last day to 0.1 g/kg of body mass to ensure gastrointestinal distress [50].

The ingestion of beta-alanine could also be beneficial in combat sports. It has been proved that using beta-alanine supplements increase intramuscular carnosine levels. Carnosine is a dipeptide prolifically found in skeletal muscle, composed of histidine and beta-alanine. Carnosine is believed to exert an important acid-base regulation function [3]. After ingestion, carnosine is degraded into histidine and beta-alanine so that it doesn't increase skeletal muscle carnosine levels [42]. On the other hand, β-alanine supplementation increases intramuscular carnosine levels. Supplementation with 1.6–6.4 g/day for at least 4 weeks significantly increases muscle carnosine content by 40–80% in training population [51]. Recent studies suggest higher efficacy with doses up to 12 g/day [52]. Beta-alanine ingestion may improve anaerobic threshold meaning that endurance activities can be performed for longer time at higher intensities [53]. Furthermore, beta-alanine ingestion may improve neuromuscular fatigue, increase lean body mass and training volume [5]. In one of the studies, 4-weeks beta-alanine supplementation significantly enhanced performance in the Special Judo Fitness Test-followed simulated combats [54].

Probably the most effective dietary supplement to increase high-intensity exercise performance in combat sports is creatine. Injecting creatine supplements can increase intramuscular phosphocreatine (PCr), which is an energy buffer protecting the ATP concentration [55]. Muscle PCr depletion during intensive training is an important factor causing fatigue. Increasing intramuscular PCr emerges can delay PCr depletion and improve anaerobic performance [3]. It has been proved that creatine supplementation increases strength, power, lean body mass and efficacy in high-intensity, short-duration exercises [56]. In Radvanovic et al. study [57], judo athletes were given 22 g of creatine monohydrate per day (in 4 doses of 5.5 g) during the first week of research in comparison to the placebo group. In the second week, subjects in the creatine group were given 1 dose of 5.5 g of creatine daily. Athletes in the placebo group followed the same protocol, but were given a dextrose solution. Before and after 2 weeks of supplementation, 30 second-Wingate cycle ergometer test was conducted on judo athletes. In creatine group the improvements in peak (12% improvement) and mean power (10,8% improvement) were 2–3 times higher in comparison to placebo group (4.4% and 5% respectively) [57]. The most common dosing protocol is creatine loading. During the first, loading phase subjects ingest high doses of creatine (approximately 20 g of creatine or 0.3 g/kg per day) in 4 doses for 5 days. In the second, maintenance phase athletes are given smaller amounts of creatine, generally 2–5 g daily (0.03 g/kg per day) for several weeks to months [42]. The average wash-out time of creatine in humans is 4 weeks. It has to be noticed that muscle PCr increase depends on the initial concentration of muscle PCr. High muscle PCr concentration can be reached by eating high amounts of creatine rich food such as meat and fish. The athletes with high initial concentration of PCr could not respond positively to creatine supplementation [58]. The most problematic aspect of creatine supplementation for combat sports athletes is a muscle water retention which leads to increasing body mass. Athletes who have to gain weight limits for a competition may cease creatine supplementation for approximately 4 weeks before weigh-in due to the average wash-out time.

Using supplements such as antioxidants may be beneficial due to alleviating oxidative stress and inflammation induced by training. Exercise induces enhanced production of reactive species leading to modifications of lipids, proteins and nucleic acid, decreased muscular functionality, histological changes and muscular soreness [59]. During prolonged and high-intensity exercises, the potential risk of free radical formation is more pronounced [60]. The study by Chun- Chung Chou [61] has shown, that 4-days' supplementation of 2000 mg vitamin C and 1400 IU vitamin E effectively attenuated inflammatory response and exercise-induced tissue damage in elite taekwondo athletes. [61]. In another study by Samaras et all [60], authors indicated that a 2 months' supplementation of a special protein bar and tomato juice significantly improved the oxidative status in ultra-marathon runners. Nevertheless, some studies have shown that antioxidants supplementation may potentially blunt beneficial physiological adaptations induced by training such as muscle oxidative capacity and mitochondrial biogenesis [62],[63].

## 7. Adverse effects of supplements

Using supplements can be connected with adverse effects that can affect the health and sports career of the athlete [64]. Supplements can be contaminated with unlisted or banned substances such as anabolic steroids [65]. The available data suggests that almost 10–15% of supplements may contain prohibited substances [66]. Despite the unintentional injection of the prohibited substance, the athlete may be banned for using illegal substances and loose medals and records set [64]. Substances such as steroids are able to improve performance, increase the size of muscle fibers and muscle strength [67]. Nevertheless, a variety of side effects can occur when steroids are misused. The risk of using steroids include health outcomes such as cardiomyopathy, acne, altered serum lipids, hepatotoxity and swollen breast tissues in men [64],[67]. Supplements may also be used by innappriopriate patterns that can lead to hypervitaminosis or problematic interactions between ingredients. [64]. The study by Tsarouhas et al. has shown that almost 86% of recreational athletes did not check the labelling of the chosen product before using it [65].

A special group of food supplements are available as preparation for weight loss. The supposed mechanism of weight loss supplements includes an impact on appetite regulation, carbohydrate absorption, fat metabolism and energy expenditure regulation. According to current scientific knowledge, none of the available, legal dietary supplements are supported by high-quality evidence [68]. The most popular, evidence-based preparation for weight loss is ephedrine which has been prohibited by Food And Drug Administration in 2004 [69]. The study by Boozer et. all has shown that a 6-month supplementation with 90 mg of ephedrine alkaloids combined with 192 mg of caffeine daily promoted body weight and fat reduction in comparision to the placebo group [70]. Despite the possitive effect on weight reduction, using ephedrine is associated with high risk of cardiovascular, gastrointestinal and psyhiatric symptoms [71]. Another potential effect of using supplements for reducing weight is hepatoxity. The supplements including garcinia cambogia, Herpalife products, green tea extract, ma huang, 1,3-Dimethylamylamine may induce liver injury [72]–[74]. In the study by Sticker et al., authors indicated that consumption of Herbalife products may be connected with liver injury. The possible reason of hepatoxic potential of the preparation was contamination of Bacillus subtilis [75]. Another study by Tse- Ling Fong et al. as shown that Hydroxycut supplementation may a cause the liver injury, nevetheless ingredients in the preparation responsible for causing the injury remain uncertain and the further research is required [76].

## 8. Reducing weight

In most combat sports, athletes are classified in weight divisions according to their body mass. Most athletes tend to reduce body weight in order to qualify for a lighter weight division so that they could fight with lighter, smaller and weaker opponents [77]. Weight loss may be achieved through chronic strategies or rapid weight loss. Rapid weight loss includes the protocol when the body mass is achieved in a few days prior to weigh-in. Both strategies could impact on sports performance and provide negative health outcomes. Chronic weight loss is mostly associated with the energy deficiency and could negatively affect on immune function, metabolic rate, bone health, hormone process and lean body mass [78],[79]. Studies have shown that weight cutting may influence testosterone level, cortisol, growth hormone, sex hormone-binding globulin and insulin. The influence on hormone process can result in decreasing bone mineral density, adolescent development and interfere blood glucose regulation [1]. The preferable energy deficiency during chronic weight loss should consitute up to 20% of energy reqiurements [9]. Kordi et al. [80] have shown that gradual weight reduction is more than twice less common than rapid weight loss in the wrestlers community. Nevertheless, gradual weight loss is more preferable [79]. If strictly necessary, rapid weight loss can be the chosen way to reduce body mass but it shouldn't exceed 5% of body mass. Furthermore, it may be reflected only if the time from weight-in to competition is sufficient to refeed and rehydrate (more than 3 hours) [3].

The most popular method of acute weight loss is an increase in exercise and restriction of fluid and food intake. To reduce weight, many athletes use dehydration protocol in order to remove excess water from the body [81]. One of the methods of reducing total body water is fluid restriction in relation to normal daily losses. Reduction of water in the body can be derived from extracellular and intracellular stores meaningly free and bound water (in glycogen stores and intestinal space) [79]. The loss of the body water is induced by urination, respiration and perspiration. Urination, as the primary method of regulating water balance is controlled by the renal system. The main hormones affecting fluid regulation are aldosterone and antidiuretic hormone-vasopressin [82]. One of the methods used by combat sports athletes to increase urine production is water loading. This technique involves the consumption of large fluid volume for several days prior to restriction. Reale at al. [81] have demonstrated that the decrease of body mass in the water loading group was higher (3.2%) in comparison to the control group (2.4%). The fluid intake in the water loading group was 100 ml/kg for 3 days in comparison to 40 ml/kg in the control group. On the 4^th^ day both groups were consuming 15 ml/kg of fluids and were following the same rehydration protocols on 5^th^ and 6^th^ days. Measurements of urea and serum electrolytes didn't exceed reference levels in both groups. There were no differences in physical performance between groups [81]. According to this protocol, water loading seems to be safe method of rapid weight loss in combat sports. In contrast to water loading, there are many popular methods of dehydration amongst combat sports athletes affecting negatively on health and sports performance. Many sportsmen in order to lose body mass use pharmacological diuretics, saunas, hot water or train in plastic suits which lead to an increase in serum osmolality, body heat storage and heart rate [79]. In the study by Amatori et al. 83] authors indicated that more severe weight loss strategies including training in heat environment, hydric restriction, using plastic wear and saunas were practiced by professional fighters and high-level athletes.

The effects of dehydration can be enhanced by using a low sodium diet. It has been proved that a high intake of sodium leads to an increase of fluid retention. Reducing the intake of sodium several days prior to weigh-in may intensify weight loss [79]. Another effective method of losing weight in combat sports is glycogen depletion [81]. Glycogen is a branched polymer of glucose stored in the liver and skeletal muscles bound to water in proportion of 1:3 g (in some cases this ratio could be higher) [84]. Glycogen storage can contribute 1–2% of skeletal muscle and up to 8% of liver weight. One of the methods leading to lower glycogen stores is a low carbohydrate diet connected with the normal training program. This protocol guides to glycogen depletion while preventing muscle glycogen restoration. Another method of degrading glycogen reserves is performing additional exercise in order to deplete glycogen stores more rapidly [8]. It has been proved that intake less than 50 g of carbohydrates daily combined with a small reduction in energy in the fight week may facilitate 1–2% of body mass loss [79]. Furthermore, it has to be noticed that decreased levels of muscle glycogen may affect negatively on isometric strength, power, endurance and time to fatigue [85]. Nevertheless, the studies by Lambert et al. [86] have shown that low carbohydrate diet lasting 2 weeks didn't impair the Wingate performance test. Similarly, Symons and Jacobs [87] reported that 2 days of a low carbohydrate diet didn't result in any changes in strength and endurance in comparison to the normal carbohydrate intake. It seems to be crucial to replenish depleted glycogen stores as soon as possible when the time to weigh-in is sufficient [79]. Another dietary strategy to reduce body mass before competition is reduction of gut contents. In order to manipulate the intestinal contents without decreasing sports performance athletes may reduce total food volume and fiber intake. Consumption of high-energy dense food with a low amount of fiber can increase body mass losses. It's proved that in comparison to high fiber diets, 2 days of low fiber intake helps to cleanse the bowel. Less than 10 g intake of fiber daily is known to be as effective as a way to reduce body weight without affecting performance [79].

## 9. Conclusions

The appropriate use of nutrition can enhance sports performance, post-workout regeneration and improve body composition in combat sports athletes. The type, as well as the time of macronutrient intake, may enhance muscle protein synthesis, recovery and tissue repair. The primary source of energy for combat sports athletes should be carbohydrates in order to ensure the proper level of muscle glycogen, which is linked with higher ability to continue moderate and high-intensity trainings.

The positive effect of a balanced diet can be enhanced by sports supplements. There are some supplements which have a proven influence on strength, endurance and body composition in combat sports such as sodium bicarbonate, caffeine, creatine, beta-alanine or protein supplements.

In most combat sports athletes tend to reduce body mass in order to obtain classification in a lighter weight division. The most preferable way to reduce weight is gradual strategy of fat reduction including low energy deficiency. If necessary, another method of reduction that may be used is rapid weight loss. It has to be noticed that acute weight loss shouldn't exceed 5% of body mass. Otherwise, it could lead to serious health problems, for instance cardiovascular and endocrine system problems or even death. Both strategies of weight loss could decrease sports performance so it's necessary to use safe, evidence-based protocols in order to minimize negative health outcomes. In order to lower the health problems and maximise sport potential, each athlete should be placed under a medical team including doctors and nutritionists. Furthermore, it's crucial to educate athletes about proper nutrition and weight loss in combat sports.
